# Structural water engaged disordered vanadium oxide nanosheets for high capacity aqueous potassium-ion storage

**DOI:** 10.1038/ncomms15520

**Published:** 2017-05-23

**Authors:** Daniel Scott Charles, Mikhail Feygenson, Katharine Page, Joerg Neuefeind, Wenqian Xu, Xiaowei Teng

**Affiliations:** 1 Department of Chemical Engineering, University of New Hampshire, Durham, New Hampshire 03824, USA; 2 Division of Chemical and Engineering Materials, Oak Ridge National Laboratory, Oak Ridge, Tennessee 37830, USA; 3 Juelich Centre for Neutron Science, Forschungszentrum Juelich GmbH, 52425 Juelich, Germany; 4 Division of X-ray Science, Advanced Photon Source, Argonne National Laboratory, Argonne, Illinois 60439, USA

## Abstract

Aqueous electrochemical energy storage devices using potassium-ions as charge carriers are attractive due to their superior safety, lower cost and excellent transport properties compared to other alkali ions. However, the accommodation of potassium-ions with satisfactory capacity and cyclability is difficult because the large ionic radius of potassium-ions causes structural distortion and instabilities even in layered electrodes. Here we report that water induces structural rearrangements of the vanadium-oxygen octahedra and enhances stability of the highly disordered potassium-intercalated vanadium oxide nanosheets. The vanadium oxide nanosheets engaged by structural water achieves high capacity (183 mAh g^−1^ in half-cells at a scan rate of 5 mV s^−1^, corresponding to 0.89 charge per vanadium) and excellent cyclability (62.5 mAh g^−1^ in full cells after 5,000 cycles at 10 C). The promotional effects of structural water on the disordered vanadium oxide nanosheets will contribute to the exploration of disordered structures from earth-abundant elements for electrochemical energy storage.

## Abstract (web summary)

The authors report that the interplay between structural water and highly disordered vanadium oxide can stabilize the layered metal oxides and enhanced their performance for aqueous potassium-ion storage based on neutron scattering measurements and electrochemical characterizations.

The shift towards clean energy utilizing alternative energy sources requires large scale electrochemical energy storage (EES) to withstand the difference between the peak hours of energy production and consumption^1^. Aqueous EES devices have several favourable characteristics over non-aqueous lithium (Li)-ion batteries; such as environmentally friendly building materials and high safety of the devices. Furthermore, they do not require strict oxygen- and water-controlled manufacturing environments and thus have much lower fabrication cost^2^. In particular, aqueous EES devices using potassium (K)-ions as charge carriers are an interesting alternative to the current Li-ion systems. The electrochemical potential of K is almost identical to that of Li, while the abundance of K in the earth's crust is close to sodium (Na) and is nearly 1,000 times greater than Li. Moreover, compared with Li- and Na-ions, hydrated K-ions possess the smallest Stokes radius (total radius of ion and bound water molecules) and great transport properties in aqueous electrolytes. Although there are reported studies on the development of new electrode materials for aqueous K-ion EES^3^,^4^,^5^,^6^, much less attention has been paid to this type of rechargeable EES system compared to those using Li- and Na-ions. This is because the large ionic radii of the K-ion causes large distortions of host electrode materials during insertion/extraction processes and leads to irreversible pulverization of the electrode upon cycling. Therefore, implementation of K-ion EES hinges on the development of electrode materials with high storage capacity and good structural stability over prolonged cycles.

The capacity and cyclability of K-ion EES can be improved through the design of layered electrode materials with large interlayer distance, so that insertion/extraction of cations with larger ionic radii can be sustained without considerable structural degradation. Among the numerous layered transition metal oxides, vanadium oxides (V_2_O_5_) with large interlayer spacing and vanadium's wide range of valance states show great promise for the storage of Na- and K-ions^7^,^8^,^9^,^10^,^11^,^12^,^13^, as well as multivalent ions such as Mg-ions^14^,^15^,^16^. Attempts to improve the capacity of vanadium-based EES include combining V_2_O_5_ with conductive materials, such as carbon black, carbon nanotubes, graphene and conductive polymers^3^,^11^,^17^,^18^, as well as the use of amorphous V_2_O_5_ with an abundant open framework that has lower entropic energy associated with the ordering of the intercalated Na-ion compared with the crystalline forms^19^.

More recently, studies have found that disordered electrodes have demonstrated great potential for enhancing the storage capacity and cycling life of EES systems. For close-packed metal oxide electrode system, electrodes with disordered cation or anion sites, accompanied accordingly with the changes of cation/anion vacancies and partial reduction of transition metal, have shown much enhanced storage capacity in Na_1.25+x_V_3_O_8_, VFe_2_O_x_ and LaMnO_3_ electrode materials^20^,^21^,^22^,^23^,^24^. Ceder's group reported that cation-disordered Li_1.2_Mo_0.47_Cr_0.3_O_2_ compounds, in which Li, Mo and Cr are disordered within the transition metal layer while the lateral stacking is well maintained and oxygen stacking is also well maintained in ABCABCABC sequences, have shown a modified local environment for the transition metal ions, and therefore provided a lower percolation threshold of Li-ions transport so that Li-ion can diffuse easily without directly contacting the transition metal framework on the atomic scale^22^. For the layered metal oxide electrode materials, turbostratic disorder has been often observed, where the metal-oxygen (M–O) layers are stacked nearly randomly on top of each other and the oxygen stacking sequences, very different from the close-packed electrode systems mentioned above. Although turbostratic disorder has been attributed to the improved storage capacities for Li- and Na-ions^12^,^25^, the stability of such disordered electrodes and their corresponding cycling life are still unsatisfactory. Therefore, improving the stability of highly disordered layered electrode materials on the charge–discharge processes without compromising the capacity and rate performance, is of great significance for EES. It becomes even more critical for K-ion storage as the large ionic radius of K causes significant distortions of the host electrode material compared to Li- and Na-ion storage.

In this study, we report that the interaction between potassium-intercalated disordered vanadium oxide (KVO) nanosheets and the structural water within the interlayer not only stabilize the KVO, but also enhance the electrochemical performance of the disordered nanosheets, evident from *ex situ* and *in situ* neutron and X-ray scattering measurements. Owing to the synergistic effects of the structural water and highly disordered nature, the KVO nanosheets demonstrate a superior K-ion storage capacity of 183 mAh g^−1^ in half-cell tests, corresponding to a striking 0.89 electron charge per vanadium atom, as well as nearly 100% capacity retention and coulombic efficiency through 5,000 cycles in full-cell tests.

## Results

### Synthesis and characterization of KVO

**Figure 1:**
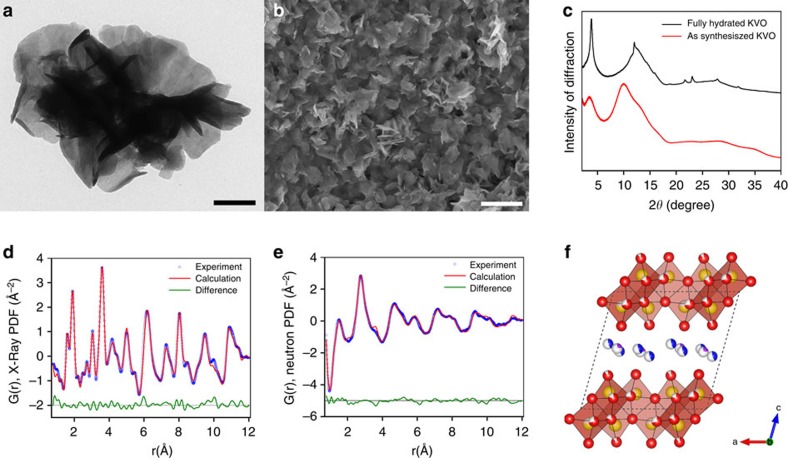
Structural analysis of disordered KVO nanosheets. (**a**) TEM image of entangled cluster nanosheets with a 200 nm scale bar. (**b**) SEM image of a drop casted coating of nanosheets with a 2 μm scale bar. (**c**) Synchrotron XRD spectra of the fully hydrated disordered KVO nanosheets and as-synthesized KVO (*λ*=0.72768 Å). PDF refinement of both (**d**) X-ray and (**e**) neutron scattering experiments; (**f**) the local structure of the disordered KVO nanosheets obtained from the refinement of X-ray and neutron PDFs, where the vanadium atoms are yellow, the potassium atoms are purple, the oxygen atoms from the V-O bilayers (O_V_) are red, the oxygen atoms from the water molecules (O_W_) are blue and vacancies are grey.

**Figure 2:**
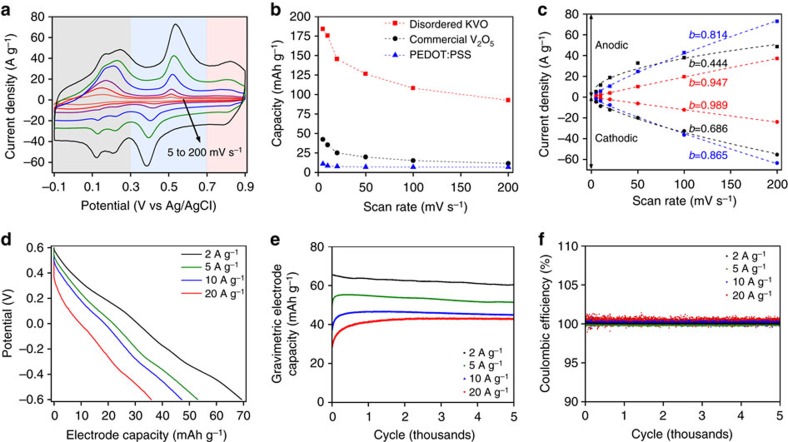
Electrochemical K-ion storage properties of disordered KVO. Electrochemical test in (**a**–**c**) half-cell and (**d**–**f**) symmetric full-cell configurations. (**a**) CV curves at various scan rates. (**b**) Calculated gravimetric electrode capacity as a function of scan rate. (**c**) Electro-kinetics analysis of the charge storage mechanisms. (**d**) Galvanostatic discharge curves at various current densities for the 500th cycle. (**e**) The average gravimetric electrode capacity through the 5,000 charge/discharge cycles. (**f**) Coulombic efficiencies as a function of cycle.

The disordered vanadium oxides were synthesized via a facile wet chemistry approach developed previously^26^, involving a reaction between potassium hydroxide with vanadyl sulfate in an aqueous solution at room temperature. Potassium hydroxide was chosen as the reagent not only to react with the vanadium precursor to form vanadium oxide, but also to allow for the inclusion of K-ions in the final product. Thereby creating crystallographic locations where K-ions can fit during the later electrochemical process. The synthetic products were thermally treated at 150 °C in air for 2 h. The resulting KVO material was soaked in de-ionized (DI) water for 2 weeks to fully hydrate before the electrochemical testing. Figure 1a,b show the transmission electron microscope (TEM) and scanning electron microscope (SEM) images of the KVO material, in which an entangled nanosheet morphology can be found with planar dimensions on the order of hundreds of nanometres. Energy dispersive X-ray spectroscopy showed that potassium was incorporated within the vanadium oxide during the synthesis, at a ratio of 0.11:1 potassium to vanadium (Supplementary Fig. 1). Synchrotron X-ray diffraction (XRD) revealed that as-synthesized vanadium oxide material is not amorphous, but poorly crystalline. Compared with the as-synthesized product, the final KVO product after thermal treatment at 150 °C and 2-week hydration process appears slightly more crystalline, but still a highly disordered material with only a few broad Bragg diffraction peaks throughout a wide range of diffraction angles (Fig. 1c). The XRD was able to establish the existence of layered structure evidenced by a strong diffraction peak at a low 2*θ* angle of 3.83°, corresponding to an interlayer distance of 10.9 Å. Due to the structural disorder of the KVO, the atomic pair distribution function (PDF) was used to investigate the local structure through neutron and X-ray total scattering experiments, as shown in Fig. 1d,e (the detailed atomic structure of KVO can be found in Supplementary Table 1). The refinement of the PDFs indicated a bilayer structure made of [VO_6_] octahedral units with both water molecules and K-ions intercalated between the V–O layers (Fig. 2f). The enlarged atomic displacement parameters of the vanadium in the direction of layered stacking (*c* axis) was observed in the refinement of the PDF, and the goodness of fit drastically decreased when the fitting range changed from intra-bilayer to inter-bilayer (at ∼15 Å; Supplementary Fig. 2). These results strongly suggest a turbostratic stacking of the vanadium oxide bilayers^27^. The PDF refinement also revealed both vanadium and oxygen vacancies, yielding the precise chemical formula of K_0.22_V_1.74_O_4.37_·0.82 H_2_O.

### Electrochemical studies of the KVO

The electrochemical K-ion storage properties of the disordered KVO nanosheets were investigated using cyclic voltammetry (CV) measurements in a three-electrode half-cell with a 1 M KCl electrolyte (Fig. 2a). A conductive ink comprised of 5 μg of the disordered KVO nanosheets and 1.25 μg of a conductive polymer, poly 3, 4-ethylenedioxythiophene-poly styrenesulfonate (PEDOT:PSS) was deposited on the surface of working electrode. The disordered KVO nanosheets exhibited excellent gravimetric capacity of 183 mAh g^−1^ at a scan rate of 5 mV s^−1^ (Fig. 2b), considerably higher than that of the highly crystalline commercial V_2_O_5_ material. Note that the contribution of PEDOT:PSS to the overall capacity is almost negligible. Given the theoretical capacity of K_2_V_2_O_5_ is 205 mAh g^−1^ based on one electron transfer per vanadium atom, the reported capacity is equivalent to 0.89 electron transfer per vanadium atom. Even at a high scan rate of 200 mV s^−1^, the disordered KVO retained a significant amount of the capacity (93 mAh g^−1^), equating to a 0.45 electron transfer per vanadium atom.

The CVs of the disordered KVO nanosheets contain multiple redox peaks at various potentials across the entirety of the 1-V potential window, as shown in Fig. 2a, during both the anodic scans (0.24, 0.51 and 0.82 V) and the cathodic scans (0.12, 0.43 and 0.79 V), indicating strong potential-dependent charge-storage mechanisms. Assuming the peak current (*i*) obeys the power law relationship with the scan rate (*v*) at a peak potential, and can be expressed as a combination of surface-controlled capacitive effects (*i*_*1*_=a_1_*v*) and diffusion-controlled K-ion intercalation (*i*_*2*_=a_2_*v*^½^):


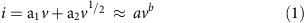


By fitting the value of *b* from equation (1), insight into the K-ion storage process can be provided^28^. Figure 2c shows the results for fitting the *b*-value of the peaks in all three of the colour coded sections of the anodic and cathodic scans. In the potential range from −0.1 to 0.3 V (black) the K-ion storage in the KVO is dominated by a diffusion-limited redox process (*b*_anodic_=0.44, *b*_cathodic_=0.69). It is worth noting the difference in mechanism for the cathodic and anodic scans in this potential range; both are closer to the diffusion-limited redox process, however the oxidation process has slower kinetics than the reduction process. In the potential range from 0.7 to 0.9 V (red) the charge storage process is controlled by surface-related capacitive process (*b*_anodic_=0.95, *b*_cathodic_=0.99); while in the potential range from 0.3 to 0.7 V (blue) both the surface-controlled capacitive and diffusion-limited redox processes contribute to the charge storage (*b*_anodic_=0.81, *b*_cathodic_=0.87). The electro-kinetic analysis suggests that charge storage of the KVO benefits from both capacitive and diffusion-limited redox processes. The former process allows the high-rate performance and the latter allows the high capacity performance of K-ion storage in the disordered KVO nanosheets. The contribution of capacitive process (double-layer capacitance and/or pseudo-capacitance) and diffusion-limited redox process to the overall capacity can be quantified with the infinite sweep rate extrapolation, as shown in Supplementary Fig. 3. Notably, at a scan rate of 5 mV s^−1^, 54% of the total capacity is attributed to diffusion-limited redox process, whereas 7% of the total capacity is attributed to redox process at 200 mV s^−1^.

We also conducted CVs in a 0.1 M Na_2_SO_4_ electrolyte (Supplementary Fig. 4). A much lower capacity is calculated and a lack of features is observed in the CV curve for Na-ion storage compared with that of K-ion storage. In the Na-ion system, the low potential redox feature ∼0.1 V completely disappears, while the redox feature at 0.4 V remains but was slightly shifted to lower potential in the anodic scan and higher potential in the cathodic scan. The results strongly confirm that the CV feature at lower potential range (−0.1 to 0.3 V) results from a diffusion-limited redox process, which is the intercalation/deintercalation of alkali between KVO layers. Although KVO has a large interlayer distance (10.9 Å), it is only sufficient for the insertion/extraction of hydrated K-ion that have much smaller Stokes radius (1.25 Å) than the hydrated Na-ion (1.87 Å). On the other hand, the CV feature at higher potential range (0.3–0.9 V) results from surface-dominated capacitive process, for which both K- and Na-ion show active storage behaviours. These results not only confirm the results from electro-kinetics analysis that diffusion-limited redox and capacitive processes for K-ion storage happen at distinct potential range, but also demonstrate the supremacy of K-ions as charge carriers for aqueous storage, in which the smaller size of hydrated K-ions are easily accommodated by the layered host materials.

Our results also show that the structural water has a significant effect on the capacity and redox behaviour of the KVO towards K-ion storage. We compared CVs of the fully hydrated material (the sample prepared by a standard procedure described above) and partially hydrated material (the sample without soaking in water after thermal treatment) in half-cell tests (Supplementary Fig. 5). The CV data of fully dehydrated sample were not included, since sample preparation requires elevated temperature, which in turn increased crystallinity from highly disordered to highly crystalline materials (XRD data are shown later). The fully hydrated material, with a chemical formula of K_0.22_V_1.74_O_4.37_·0.82 H_2_O, was prepared by soaking thermally treated materials in water for 2 weeks before CV tests. Conversely, the partially hydrated material, with a chemical formula of K_0.22_V_1.74_O_4.15_·0.46 H_2_O was directly used to prepare an ink for CV tests after thermal treatment. As shown in Supplementary Fig. 5, a clear increase in the redox features of the CVs in half-cell measurements is observed in fully hydrated KVO. Specifically, the capacity of fully hydrated KVO showed a significant increase of 41% (from 103 to 145 mAh g^−1^) compared with partially hydrated material at a scan rate of 20 mV s^−1^, and a less but still considerable increase of 19% (from 78 to 93 mAh g^−1^) at 200 mV s^−1^.

The long-term cyclability of KVO towards K-ion storage was tested in a symmetric full-cell configuration in a 3 M KCl electrolyte at various current densities ranging from 2 to 20 A g^−1^. Figure 2d shows the nearly linear potential-capacity curves. The calculated electrode capacities are shown as a function of cycle in Fig. 2e. It can be clearly seen that there is no obvious capacity decay through the 5,000 charge–discharge cycles at all the current densities tested, demonstrating excellent capacity retention. Analogously, the coulombic efficiencies (Fig. 2f) of the KVO full-cell, the ratio of the amount of charge taken from the device to the amount stored during each cycle, are nearly 100% throughout the 5,000 cycles. It is important to point out that at 2 A g^−1^, corresponding to an average discharge time of ∼360 s (equivalent to a C-rate of 10), KVO cells demonstrate an electrode capacity of 62.5 mAh g^−1^ after 5,000 cycles, corresponding to a 0.31 electron transfer per vanadium atom. Even at 20 A g^−1^ (corresponding to an average C-rate of 1,800), KVO cells still demonstrate an electrode capacity of 40 mAh g^−1^ after 5,000 cycles (0.2 electron transfer per vanadium). We notice that electrode capacities obtained from full-cell cycling were lower than those from CV measurements in half-cell, which may be largely due to the fact that active material loading in full-cell was around 700 times higher than that in half-cell (3.5 mg versus 5 μg).

The long-term cyclability of partially hydrated and the highly crystalline KVO materials was also tested in comparison to that of fully hydrated disordered KVO as shown in Supplementary Fig. 7, the trend of capacity following the order of fully hydrated KVO>partially hydrated KVO>crystalline KVO. The partially hydrated materials were prepared after thermal treatment at 150 °C for 2 h without soaking in the water for 2 weeks, while the crystalline materials containing no structural water were prepared by thermal treatment at 400 °C for 2 h as shown in Supplementary Fig. 6. The results show that the crystalline materials exhibits a much lower capacity to those of the fully and partially hydrated disordered KVO materials. We also notice that the capacity of the partially hydrated materials is significantly lower than that of the fully hydrated material in the initial cycles, and continuously increases through cycling. The results indicate that the repeated intercalation/deintercalation process of the K-ions during cycling, likely accompanied with water molecules, appears to induce a hydration process which steadily modifies and stabilizes the structure of the partially hydrated KVO further, closer to that of the fully hydrated material.

The results from Supplementary Fig. 7 also showed that discharge capacities of KVO materials, including fully hydrated, partially hydrated and crystalline materials, increased rather rapidly in the initial cycles. For fully hydrated materials, the capacities reached the maximum values at ∼1,000 cycles before showing a minor but steady fade for the rest of cycles. However, for partially hydrated and crystalline materials, the capacities showed less drastic but still continuous increases even after ∼1,000 cycles. The exact mechanism of the increasing capacity especially during the initial cycling, is still unclear. However, such ‘conditioning behaviours' may result from a hydration process, from which the electrode reach its optimal electrochemical condition for better performance. The longer ‘conditioning period' observed in partially hydrated and crystalline KVO (nearly throughout entire 5,000 cycles) again suggested that the hydration may be one of the most important outcomes of the ‘conditioning process', whereas the fully hydrated KVO indeed required less ‘conditioning process'. However, it has been pointed out that even if cycling induced hydration improves capacity for the partially hydrated and crystalline materials dramatically, it is still possible that other degradation processes may occur.

### Structural water effect

**Figure 3:**
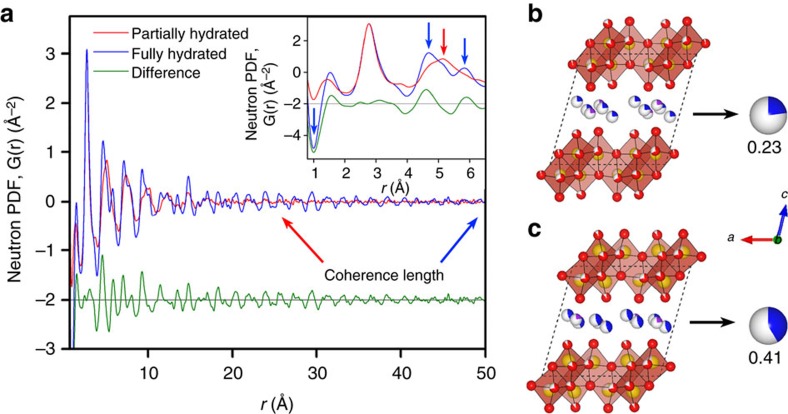
Neutron PDFs of partially and fully hydrated disordered KVO. (**a**) Experimentally collected neutron PDFs of partially and fully hydrated disordered KVO. The fully hydrated material showed much larger coherence length. The inset shows the rearrangement of the local structure on hydration. The refined structures and occupancy of the water intercalated between the layers as quantified by fitting PDF analysis for the (**b**) partially hydrated KVO has an O_W_ (the oxygen atoms from the water molecules, blue) occupancy of 0.23 between V–O layers, while (**c**) hydrated KVO has an O_W_ occupancy of 0.41. In the refined local structure of KVO materials, the vanadium atoms are yellow, the potassium atoms are purple, the oxygen atoms from the V–O bilayers are red and the vacancy sites are grey.

To elucidate the mechanisms of high capacity and high stability found in the disordered KVO nanosheets, we conducted neutron-based PDF analyses to study the influence of structural water on structural stability of disordered KVO. Neutron PDF was used due to the high sensitivity of neutrons to water (being particularly sensitive to both oxygen and hydrogen atoms) compared with X-ray and electron probes. Additionally, the lack of long range order of the KVO nanosheets and intercalated species (for example, water and K-ion) required the utilization of PDF analysis so that the effect of hydration on local structure can be studied. Our neutron scattering experiments and PDF analysis showed that on hydration the increase in structural water of the KVO material was also accompanied by re-ordering the local structure and an increase of the coherence as shown in Fig. 3a. The refined structures of the partially hydrated and fully hydrated disordered KVO are shown in Fig. 3b,c. They possess a similar monoclinic structure having potassium and water intercalated within V–O bi-layers consisting of [VO_6_] octahedral units. Albeit, a much larger coherence length is observed in the fully hydrated KVO (∼50 Å) than that of partially hydrated KVO (∼28 Å). This enhanced coherence is still on the order of single nanometres and much smaller than that of a highly crystalline V_2_O_5_ bulk material (Supplementary Fig. 8a). We also notice that the X-ray PDF of the fully hydrated KVO shows a similar coherence length as the neutron PDF (Supplementary Fig. 9), thus allowing us to attribute it to a property of the material rather than an artifact in the data caused by the significant amount of hydrogen in the sample.

Many changes in the local structure are observed on the hydration of the disordered KVO, including a large increase in water content evident by an increase in intensity of O–H bond (*r*=1 Å), as well as new O–O features observed in the fully hydrated KVO at a radial distance of 4–6 Å in the PDF as marked in the inset of Fig. 3a. We note that the latter difference is not only attributed to the ordering of O_W_–O_W_ (O_w_ is the oxygen atoms from structural water molecules between V–O bilayers) and O_V_–O_W_ (O_V_ is the oxygen atoms from V–O bilayers), but mainly to a change in the O_V_–O_V_ correlations. This indicates that the hydration primarily causes structural rearrangements of the [VO_6_] octahedra that make up the V–O bilayers. More details about the hydration effect can be found in Supplementary Fig. 8b,c, showing all of the different O–O contributions to the PDF models for the partially and fully hydrated material refinement results. The correlation between O_V_ and O_W_ is only a minor contributor to the neutron PDF, however the fully hydrated material has stronger correlations compared to the partially hydrated material. The fully hydrated material possesses almost double the amount of water, as quantified by the refinement and also visually evident via the difference in magnitude of the O–H correlations after normalization of the data sets. A comparison of all of the various O–O contributions for the partially and fully hydrated KVO to the PDF models, including O_V_–O_V_, O_V_–O_W_ and O_W_–O_W_, have been isolated and are shown in Supplementary Fig. 8d,e. The dominant contribution of O_V_–O_V_ to the PDFs clearly indicates that hydration of KVO nanosheets occurs along with oxidation and structural rearrangement of the V–O bilayers and increases the ordering and coherence length of the material.

The influence of different stacking patterns on the fit of the X-ray PDF at high-r for the fully hydrated disordered KVO material has also been investigated, in which a simplified model was used to create an additional 5 models containing 2,3,4,5 and 6 independent bilayers as shown in Supplementary Figs 10–15. In this model, the V–O bilayers were shifted using least squares refinement to fit the X-ray PDF data to preserve the local structure and investigate the possibility of different stacking patterns. The results are shown in Supplementary Fig. 16, revealing that a drastic improvement in the goodness of fit (*R*_WP_) can be made by increasing from 1 to 4 bilayers through introducing only minor shifts of V–O bilayers. Further, smaller but still continuous improvements are seen through adding a 5th and 6th bilayer to the model. The shifts of the V–O bilayers are minor changes to the expected position and then can be considered as disordered stacking rather than a different stacking pattern. These results lead us to believe that our assertion of turbostratic disorder accounts for the increasingly poor fit at high-r range.

### *In situ* XRD analysis

**Figure 4:**
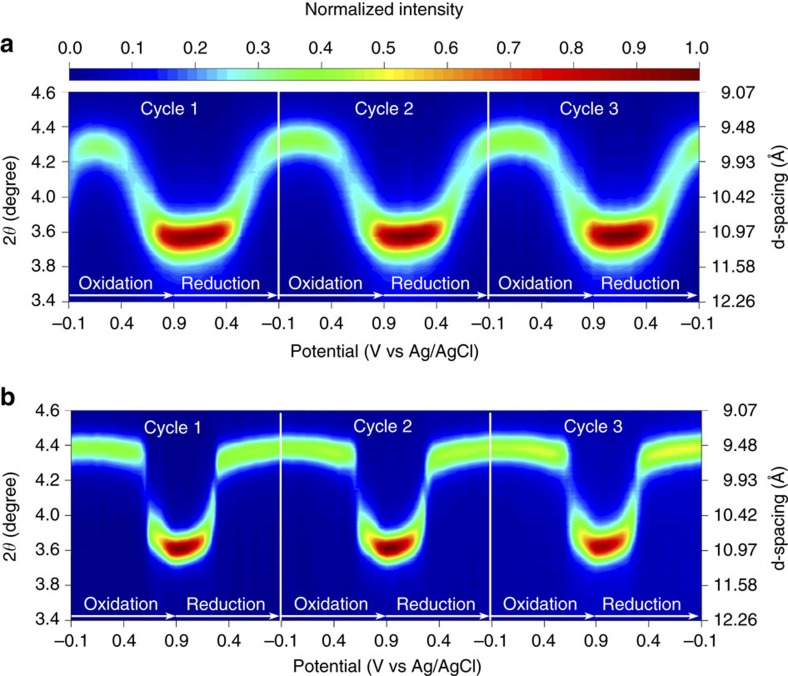
*In situ* XRD spectra of the layered (001) plane during electrochemical cycling. The contour plot of *in situ* XRD spectra during the three CV cycles at a scan rate of 1 mV s^−1^ for the (**a**) disordered KVO nanosheets and (**b**) ordered KVO materials in a 3 M KCl electrolyte.

Second, the influence of disorder on charge-transfer processes of KVO nanosheets was studied with *in situ* XRD measurements during the first three cycles of CVs at a rate of 1 mV s^−1^ (Supplementary Fig. 11). The charging (oxidation) process proceeds as the potential increases from −0.1 to 0.9 V, the (001) reflection peak of KVO shifted to lower 2*θ* angles, while at the same time the peak narrowed and increased in diffraction intensity. Inversibly during the discharge process (reduction) both the position and the shape of (001) diffraction peak of KVO were restored to those of the original state by the end of the first cycle. Nearly identical behaviours were also found in the second and third cycle. The reversible changes of the (001) peak position during the cycling, in a manner analogous to what is observed in other 2D systems such as Ti_3_C_2_ (ref. 29), can be explained by the following mechanism: when positively charged K-ions intercalated between negatively charged V–O layers, the electrostatic interaction between V–O layers increased and then caused a decrease of interplanar distance, and *vice versa*. Moreover, our data indicated that insertion of the K-ion not only strengthened electrostatic interaction between V–O layers, but also caused structural disorder. The (001) peak broadening shown in Fig. 4a indicated a wider distribution of V–O interlayer distances within a crystallite as K-ions intercalated. The *in situ* XRD patterns of the disordered KVO also contained a peak identified as the (020) diffraction plane, which shifted to higher 2*θ* angle during the oxidation process due to the increased oxidation state of vanadium and shorter V–O bond distance, and reversibly shifted back to lower 2*θ* as the vanadium was reduced and the V–O bond distance increased during the reduction process.

To further understand the synergistic effect of structural water and disordered KVO structure on promoting K-ion storage, *in situ* XRD measurement was also conducted on a highly crystalline potassium-intercalated layered V_2_O_5_ without structural water, prepared by thermal treatment of KVO at 400 °C for 2 h in air as shown in Supplementary Figs 6 and 12. The thermal treatment at the elevated temperature produces a highly ordered KVO material with rod like morphology which is also layered, but with a smaller layer–layer distance, *d*_001_=9.8 Å. The CVs of the ordered KVO are very different in shape compared to that of the disordered material. A single strong redox couple is visible in the CV curve and a much lower overall capacity. Figure 4 shows the disordered and highly crystalline KVO showed very similar *in situ* XRD patterns in terms of peak shifting and narrowing/broadening of the (001) diffraction peak. Interestingly, a significant difference between the two materials is that the (001) peak shifting of the disordered KVO occurred within much broader potential range than the highly crystalline KVO. The results strongly suggest that K-ion transport between V–O layers in the disordered structure has much lower energy barriers compared with that in the ordered structure, which is expected to expand the accessible potential window for K-ion insertion and extraction, and therefore to enhance the storage capacity. Another appealing feature is that the disordered KVO displays continuous and reversible peak shifts of the (001) reflection during K-ion extraction/insertion without obvious structure transformation, compared to the highly crystalline KVO which undergoes a more distinct staged-like peak shifting. The results indicate a high structural stability of KVO induced by the interaction between V–O layers and water, which also corroborates the excellent cycling stability and the high-rate capability in the button-cell measurements.

## Discussion

In conclusion, we have demonstrated promotional effects of structured water on stability and electrochemical performance of highly disordered K_0.22_V_1.74_O_4.37_·0.82 H_2_O nanosheets. Compared with previous work on layered materials for aqueous energy storage, we directly observed the interaction between structured water and V–O layers using X-ray and neutron scattering accompanied with the refinement of PDFs. Interplay between structural water and disordered KVO exhibits superior high capacity (0.89 electrons transfer per vanadium atom) and high rate (0.31 electrons transfer per vanadium atom at a C-rate of 10) performance for K-ion storage in an aqueous electrolyte. In addition to V_2_O_5_, our ongoing project showed that similar promotional effect of structural water can also be found in MnO_2_ birnessite nanolayers (data are not reported here). We believe that improving the stability and electrochemical performance of the disordered electrode materials through the engagement of structural water, a strategy reported in our studies, offers a new paradigm for developing various highly stable and effective disordered metal oxide electrode materials for aqueous energy storage.

## Methods

### Material synthesis

Synthesis of the disordered KVO nanosheets was conducted at room temperature in atmospheric conditions in a 50 ml magnetically stirred batch reactor vessel. Vanadyl (IV) sulfate oxide hydrate (VOSO_4_·H_2_O, Alfa Aesar, 99.9% metals basis) was dissolved in DI H_2_O at a concentration of 10 mg ml^−1^. A syringe pump was used to add a 17.3 mg ml^−1^ potassium hydroxide solution (KOH, Alfa Aesar, Pellets, 99.98% metals basis) at a rate of 0.25 ml min^−1^ over 40 min. The reaction vessel was allowed to mix for an additional 30 min before the products were centrifuged, washed with water and ethanol and vacuum dried. The dry powder was then thermally treated at 150 °C for 2 h. The post thermally treated powder was allowed to soak in water for 2 weeks. Synthesis of ordered KVO materials were conducted in an analogous way to the disordered KVO nanosheets, except the thermal treatment was conducted at 400 °C. The commercial V_2_O_5_ used was Sigma Aldrich Vanadium (V) Oxide≥99.6% trace metals basis. It has been pointed out that vanadium compounds are toxic. Particularly, an exposure limit of 0.05 mg m^−3^ for V_2_O_5_ dust and 0.1 mg m^−3^ for V_2_O_5_ fumes in the workplace, and 35 mg m^−3^ of vanadium is considered immediately dangerous to life and health.

### Structural characterizations

TEM images were collected with a Zeiss/LEO 922 Omega TEM at the University of New Hampshire. SEM images were collected using the Tescan Lyra3 GMU FIB SEM at the University of New Hampshire, an Edax attachment was used for energy dispersive X-ray spectroscopy (EDS) measurements. X-ray PDF experiments were conducted at beamline 11-ID-B at the Advanced Photon Source, Argonne National Laboratory. Neutron PDF experiments were conducted at the Nanoscale-Ordered Materials Diffractometer (NOMAD, 1B) at the Spallation Neutron Source, Oak Ridge National Laboratory. The refinement of PDF data was done with PDFgui software. The Q-maximum used for the X-ray PDF was 23.5 Å^−1^. The Q-maximum used for the Neutron PDF was 25 Å^−1^, with the Lorch function applied. The powder samples were loaded into quartz capillaries (length: 80 mm; inner diameter: 3 mm) and were exposed to the beam for various times (3 h for the hydrated disorder KVO, 2.5 h for the partially hydrated disordered KVO and 2.25 h for the bulk V_2_O_5_). Although we also prepared the sample with isotopically substituted water (D_2_O), the light water (H_2_O) contribution, possibly introduced from moisture in the air, results in overwhelming O–H interaction relative to O–D interaction.

### Electrochemical measurements

The half-cell measurements were conducted using a three-electrode electrochemical half-cell, consisting of a rotating disc working electrode (glassy carbon electrode), Ag/AgCl reference electrode and Pt wire counter electrode, using a CHI 660E Electrochemical Workstation and Pine Modulated Speed Rotator. A sample of 5 μg of active material and 1.25 μg poly(3, 4-ethylenedioxythiophene)-poly(styrenesulfonate; PEDOT: PSS), (Sigma Aldrich 3.0–4.0% in H_2_O, High Conductivity grade) was loaded on the working electrode for all materials tested. 1% Nafion (20 μl; Sigma Aldrich Nafion 117 solution) by volume in DI water was applied on the working electrode after the application of the active material. CVs were conducted in a 1 M KCl electrolyte from −0.1 V to 0.9 V (versus saturated Ag/AgCl) and scanned at various rates between 5 and 200 mV s^−1^. The second cycle of CVs was used for electrochemical analysis.

Two-electrode full-cell were fabricated by the following procedures: First, depositing 3.5 mg KVO (∼700 times more materials loaded in full-cell as compared to the half-cell measurements), 0.7 mg PEDOT:PSS and 0.7 mg PTFE (Sigma Aldrich Polytetrafluoroethylene 60 wt% dispersion in H_2_O) binder onto a Toray carbon paper with a surface area of 2.54 cm^2^. Second, the two electrodes were assembled into a symmetric configuration in EL-Cell ECC-AQU electrochemical cells. Finally, the cells were tested in a 3 M KCl electrolyte with a 1.2 V potential window (−0.6 V to 0.6 V) at various current densities ranging from 2 to 20 A g^−1^ for 5,000 cycles using an Arbin BT-G Battery Analyzer.

### *In situ* X-ray diffraction

*In situ* XRD measurements were conducted at the beamline 17-BM-B, Advanced Photon Source, Argonne National Laboratory (*λ*=0.72768 Å). The *in situ* cell was designed at The University of New Hampshire, made of polycarbonate with Kapton tape windows to allow for penetration of X-rays. CVs were conducted in the half-cell configuration with a 3 M KCl electrolyte at 1 mV s^−1^ from −0.1 V to 0.9 V (versus 3 M Ag/AgCl micro-reference electrode).

### Data availability

The authors declare that the data supporting the findings of this study are available within the paper and its Supplementary Information Files.

## Additional information

**How to cite this article:** Charles, D. S. *et al*. Structural water engaged disordered vanadium oxide nanosheets for high capacity aqueous potassium-ion storage. *Nat. Commun.* **8**, 15520 doi: 10.1038/ncomms15520 (2017).

**Publisher's note:** Springer Nature remains neutral with regard to jurisdictional claims in published maps and institutional affiliations.

## Supplementary Material

Supplementary Information Supplementary Figures, Supplementary Tables and Supplementary Notes
