# Supplementary material for: Structural water engaged disordered vanadium oxide nanosheets for high capacity aqueous potassium-ion storage
Source: Nat Commun. 2017 May 23;8:15520. doi: 10.1038/ncomms15520 (PMC5457508; doi:10.1038/ncomms15520)
Supplement: Supplementary Information — Supplementary Figures, Supplementary Tables and Supplementary Notes [file ncomms15520-s1.pdf]

Supplementary Information

Supplementary Figures

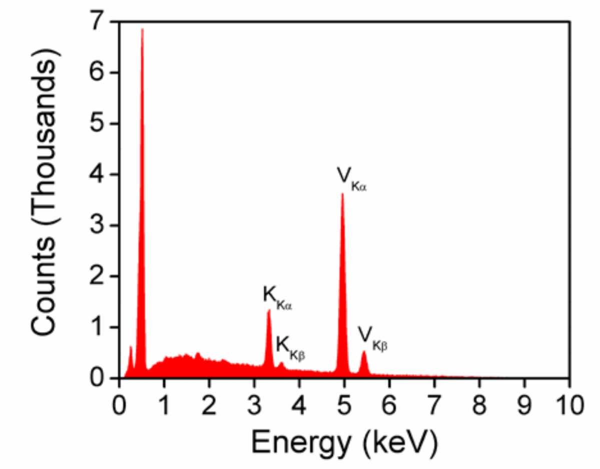

Supplementary Figure S1. Energy Dispersive X-ray Spectrosopy (EDS) measurement of the disordered KVO nanosheets. The EDS shows that after the separation, washing proccesses and thermal treatment a significant amount of potassium is detectable in the sample at an atomic ratio of 0.11 to 1 (Potassium vs Vanadium).

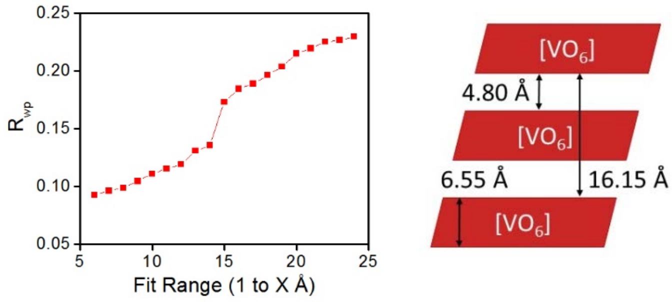

Supplementary Figure S2. Turbostratic Disorder of the disordered KVO nanosheets. (**a**) Goodness of fit (R_{WP}) of the X-ray PDF with increasing maximum value of the fit range showing a drastic decrease in goodness of fit (increase of R_{WP}) at 15 Å corresponding to the change form intra-bilayer and inter-bilayer. (**b**) Schematic of the sacking of the bilayers made up of the [VO_6] octahedral units.

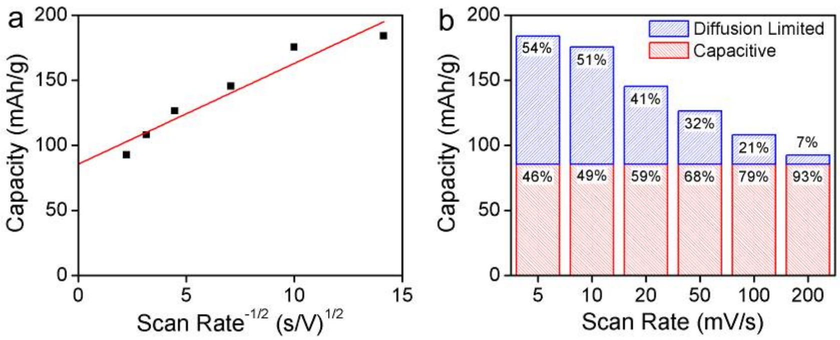

Supplementary Figure S3. Infinite Sweep Rate Extrapolation. (**a**) Fit of capacitance with square root of (1/*v*) for the disordered KVO nanosheets, where the intercept indicates the capacity at an infinite sweep rate and (**b**) a stacked bar graph showing the percent of total capacitance coming from the diffusion limited and capacitive contributions. (The capacity at infinite sweep rate is considered the capacitive contribution, which is constant.)

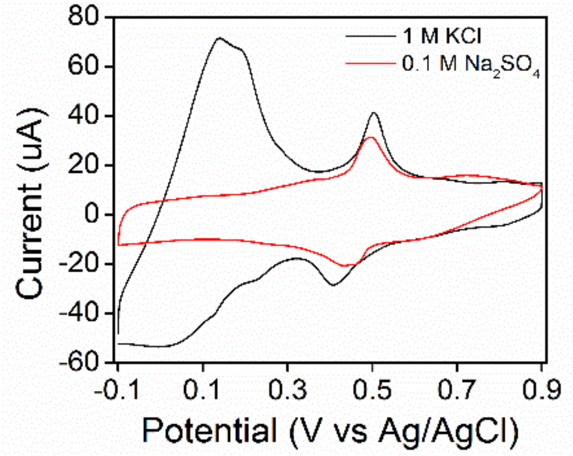

Supplementary Figure S4. CV measurements at 20 mV s^{-1} of the disorderd KVO nanosheets in a 1 M KCl and a 0.1 M Na_2SO_4 comparing the effectiveness of charge storage of K- and Na-ions in aqueous electrolytes.

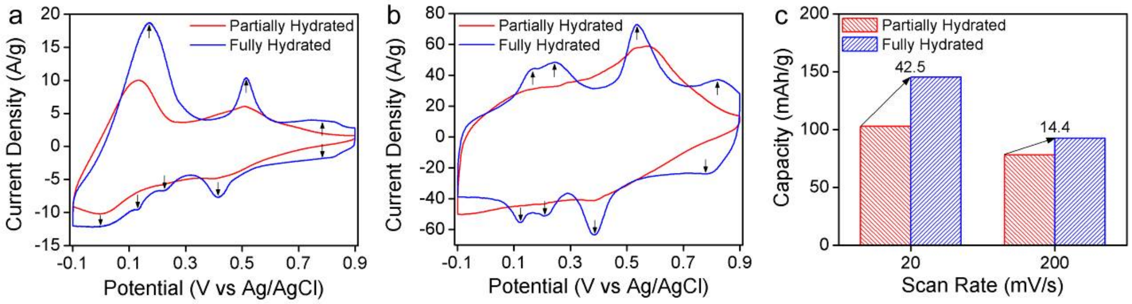

Supplementary Figure S5. Hydration effect on CVs at (**a**) 20 mV s^{-1} and (**b**) 200 mV s^{-1} and the (**c**) calculated gravimetric capacities of the partially hydrated (not soaked in water for two weeks) and fully hydrated (soaked in water for two weeks) disordered KVO nanosheets.

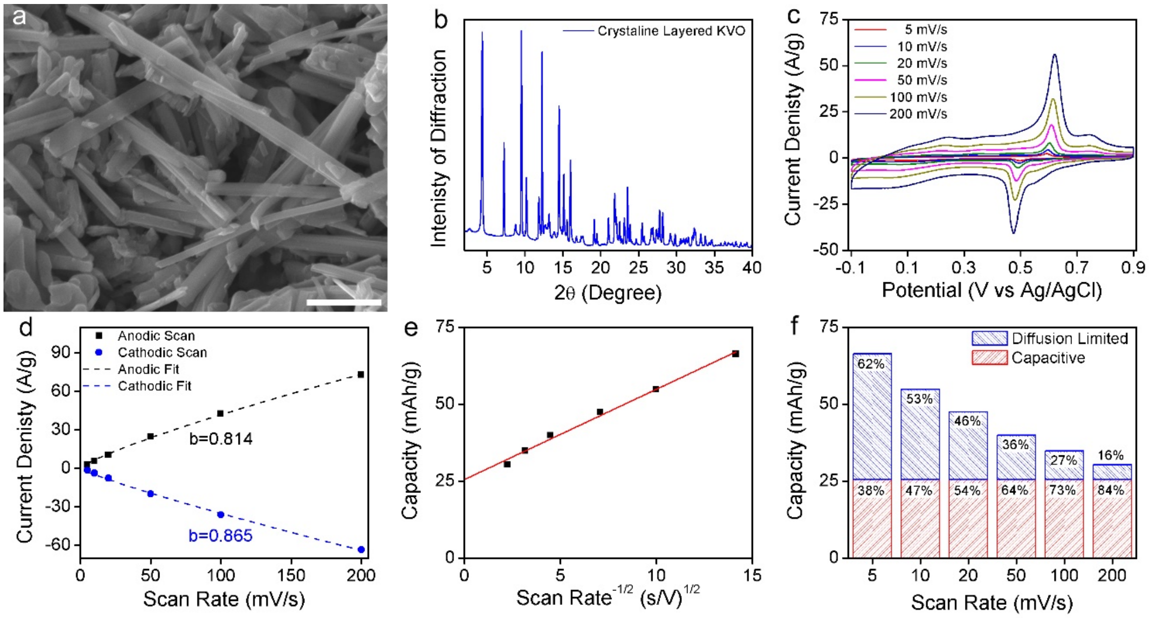

Supplementary Figure S6. Structure and Electrochemical Characterization of Crystalline/Ordered KVO. (**a**) SEM image of the ordered KVO showing the formation of rods with a 1 μm scale bar. (**b**) XRD spectra with sharp and well defined Bragg peaks showing the crystalline nature of the sample, while still exhibiting a layered crystal structure with a layered peak at low 2θ angle (4.36 2θ, d_{001}=9.56 Å). (**c**) CV measurements at various scan rates in a 1 M KCl using the three electrode half-cell. (**d**) b-value calculation for the redox peak in the anodic and cathodic scan of the CVs. (**e**) Fit of infinite sweep rate extrapolation and (**f**) a stacked bar graph showing the percent of total capacity coming from the diffusion limited and capacitive contributions.

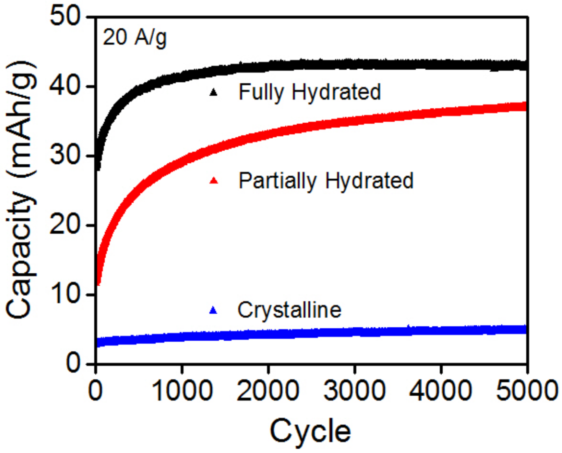

Supplementary Figure S7. The average gravimetric electrode capacities of the fully hydrated disordered KVO in comparison with the partially hydrated and crystalline KVO materials through the 5,000 charge/discharge cycles at a current density of 20 A/g.

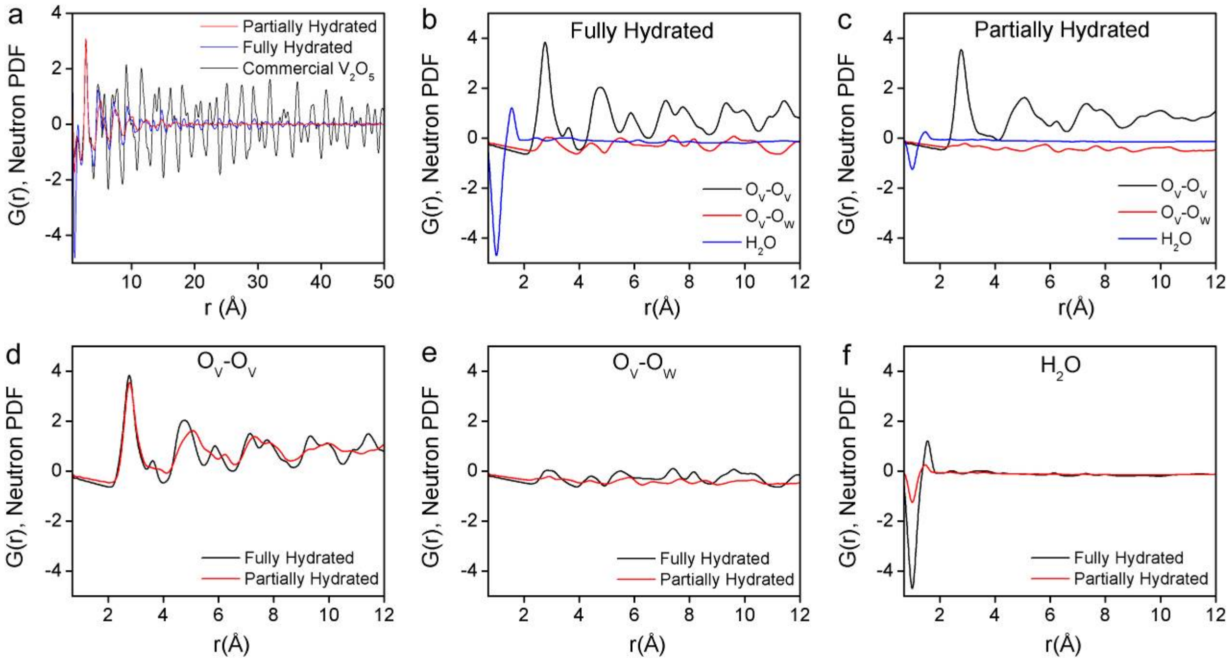

Supplementary Figure S8. The neutron PDF analysis of partially hydrated, fully hydrated KVO, as well as commercial V_2O_5. **(a)** The comparison of coherence in the Neutron PDFs of the partially hydrated (not soaked in water for two weeks) and fully hydrated (soaked in water for two weeks) disordered KVO nanosheets with Commercial V_2O_5. Oxygen correlations from fitted local structure models for the (**b**) fully hydrated sample and (**c**) partially hydrated sample. Comparison of the fully hydrated and partially hydrated sample interactions from fitted structural models (**d**) O_V-O_V (**e**) O_V-O_W (**f**) O_W-O_W. (O_V is the oxygen atoms from V-O bilayers and O_w is the oxygen atoms from structural water molecules.)

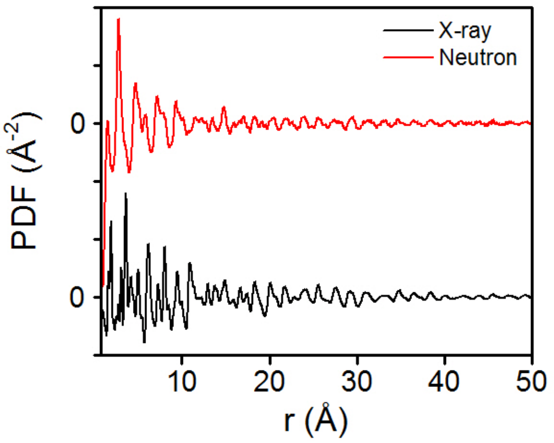

Supplementary Figure S9. The comparison of coherence in the X-ray and neutron PDFs of the fully hydrated (soaked in water for two weeks) disordered KVO nanosheets.

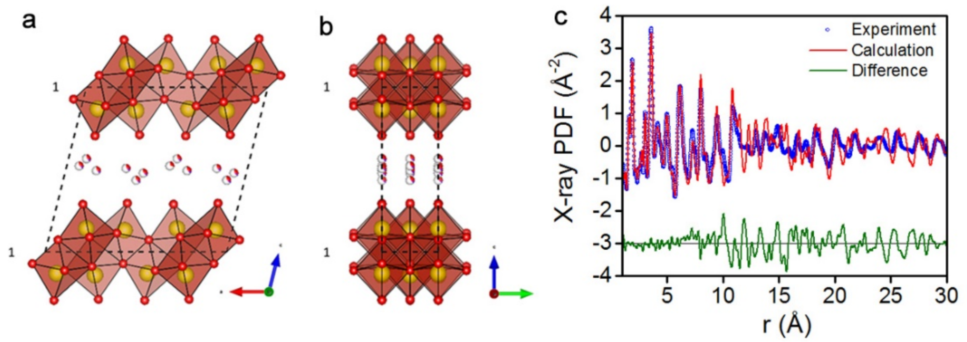

Supplementary Figure S10. A simplified model was used to construct stacking patterns containing a *single* bilayer. The local structure was fit to 6 Å using a fully isotropic, fully occupied model for the vanadium oxide bilayers in which the water (represented by oxygen) and potassium occupy the same atomic position within the layers at the appropriate occupancies to give the correct chemical composition. The stacking pattern is shown in both the (a) *ac* and (b) *bc* plane. (c) The resulting X-ray calculated PDFs and difference curves are also shown.

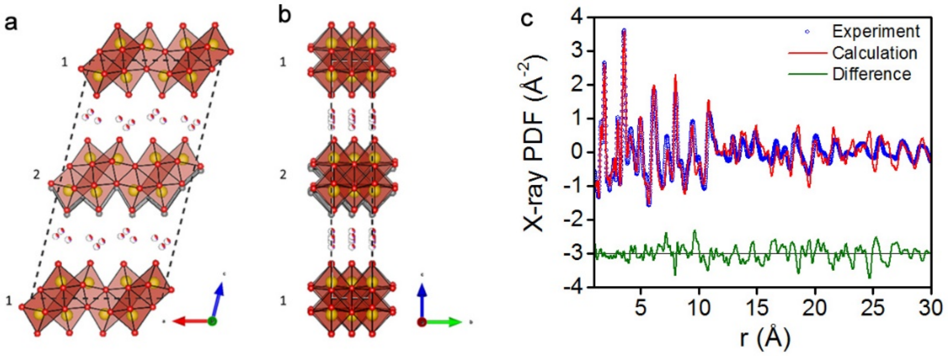

Supplementary Figure S11. A simplified model was used to construct stacking patterns containing *two* bilayers. The local structure was fit to 6 Å using a fully isotropic, fully occupied model for the vanadium oxide bilayers in which the water (represented by oxygen) and potassium occupy the same atomic position within the layers at the appropriate occupancies to give the correct chemical composition. The vanadium oxide bilayers were shifted using least squares refinement of the X-ray PDF data of the fully hydrated KVO. The stacking pattern is shown in both the (a) *ac* and (b) *bc* plane, the gray shadows are of the non-shifted model (as shown in Figure S10) which are there to highlight the shifting of the bilayers. (c) The resulting X-ray calculated PDFs and difference curves are also shown.

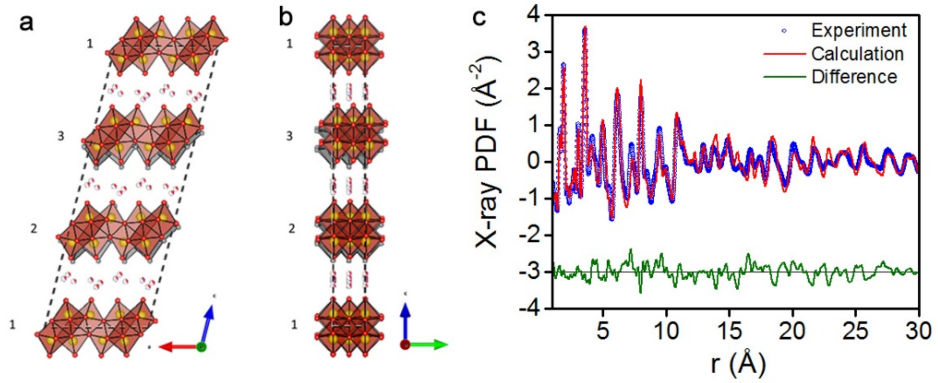

Supplementary Figure S12. A simplified model was used to construct stacking patterns containing *three* bilayers. The local structure was fit to 6 Å using a fully isotropic, fully occupied model for the vanadium oxide bilayers in which the water (represented by oxygen) and potassium occupy the same atomic position within the layers at the appropriate occupancies to give the correct chemical composition. The vanadium oxide bilayers were shifted using least squares refinement of the X-ray PDF data of the fully hydrated KVO. The stacking pattern is shown in both the (a) *ac* and (b) *bc* plane, the gray shadows are of the non-shifted model (as shown in Figure S10) which are there to highlight the shifting of the bilayers. (c) The resulting X-ray calculated PDFs and difference curves are also shown.

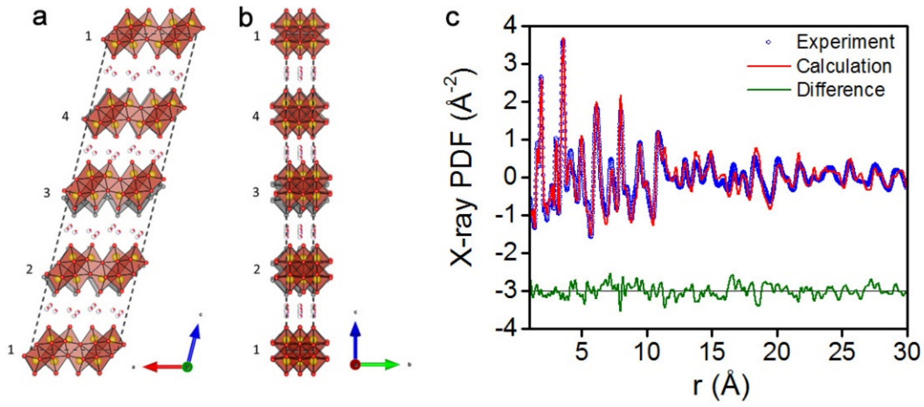

Supplementary Figure S13. A simplified model was used to construct stacking patterns containing *four* bilayers. The local structure was fit to 6 Å using a fully isotropic, fully occupied model for the vanadium oxide bilayers in which the water (represented by oxygen) and potassium occupy the same atomic position within the layers at the appropriate occupancies to give the correct chemical composition. The vanadium oxide bilayers were shifted using least squares refinement of the X-ray PDF data of the fully hydrated KVO. The stacking pattern is shown in both the (a) *ac* and (b) *bc* plane, the gray shadows are of the non-shifted model (as shown in Figure S10) which are there to highlight the shifting of the bilayers. (c) The resulting X-ray calculated PDFs and difference curves are also shown.

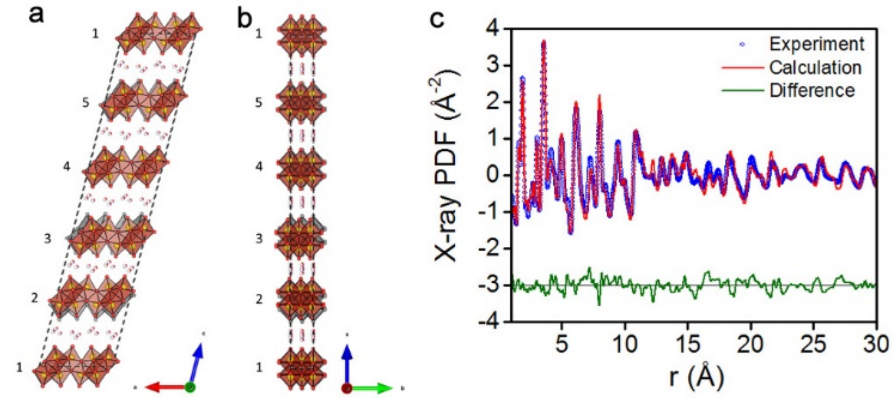

Supplementary Figure S14. A simplified model was used to construct stacking patterns containing *five* bilayers. The local structure was fit to 6 Å using a fully isotropic, fully occupied model for the vanadium oxide bilayers in which the water (represented by oxygen) and potassium occupy the same atomic position within the layers at the appropriate occupancies to give the correct chemical composition. The vanadium oxide bilayers were shifted using least squares refinement of the X-ray PDF data of the fully hydrated KVO. The stacking pattern is shown in both the (a) *ac* and (b) *bc* plane, the gray shadows are of the non-shifted model (as shown in Figure S10) which are there to highlight the shifting of the bilayers. (c) The resulting X-ray calculated PDFs and difference curves are also shown.

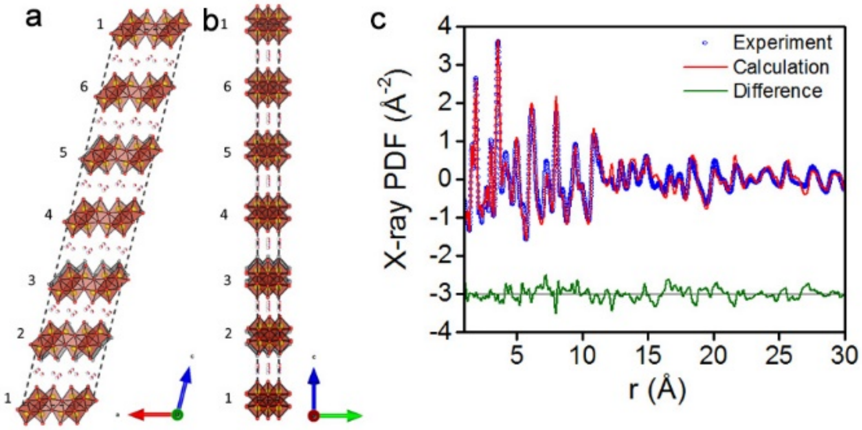

Supplementary Figure S15. A simplified model was used to construct stacking patterns containing *six* bilayers. The local structure was fit to 6 Å using a fully isotropic, fully occupied model for the vanadium oxide bilayers in which the water (represented by oxygen) and potassium occupy the same atomic position within the layers at the appropriate occupancies to give the correct chemical composition. The vanadium oxide bilayers were shifted using least squares refinement of the X-ray PDF data of the fully hydrated KVO. The stacking pattern is shown in both the (a) *ac* and (b) *bc* plane, the gray shadows are of the non-shifted model (as shown in Figure S10) which are there to highlight the shifting of the bilayers. (c) The resulting X-ray calculated PDFs and difference curves are also shown.

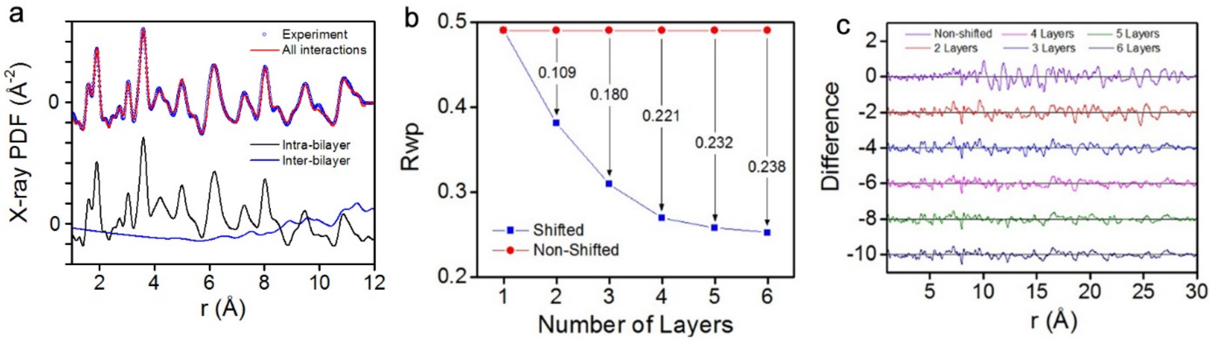

Supplementary Figure S16. Investigation of the difference between inter and intra-bilayer interactions and stacking of bilayers. (**a**) The isolated inter and intra-bilayer correlations to the fit of X-ray PDF in main text Fig. 1. Although the first inter-bilayer correlations start at 5 Å, it is not until 9 Å where the inter-bilayer correlations become more significant. Nevertheless, the shape of the distinct peaks is still due to the intra-bilayer interactions as the inter-bilayer interactions produce non distinct broad features. (**b**) The decreasing goodness of fit (R_{WP}) with increasing number of shifted layers in the different stacking models, individual fits shown in Figure S10 to S15. The increasing number of shifted bilayers, all possessing the same local structure for vanadium oxide bilayers, significantly reduced the R_{WP} with only minor shifts suggesting that there is disordered staking of the bilayers in the material rather than a different stacking pattern. (**c**) Difference curves of the stacking models with the various numbers of independently shifted bilayers. An improvement can clearly be seen in the fit of the data at high-r with increasing number of shifted bilayers, these results lead us to believe that our assertion of turbostratic disorder accounts for the increasingly poor fit at high-r range.

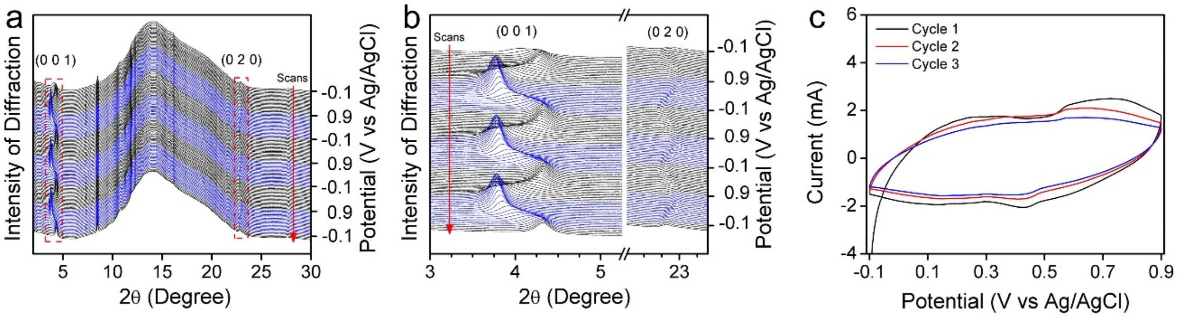

Supplementary Figure S17. *In situ* XRD of the disordered KVO nanosheets. (**a**) Full spectra of *in situ* XRD showing the large background from the aqueous 3 M KCl electrolyte. (**b**) close up of the selected regions showing the evolution of the peaks with potential through three cycles. (**c**) Three cycle CV measurement at 1 mV/s in a 3 M KCl electrolyte conducted while collecting the *in situ* XRD spectra. The *in situ* XRD spectra of the disordered KVO also contained a brag diffraction peak at 22.94° 2θ, identified as the (020) diffraction plane, that also showed a potential dependence during cycling. The (020) diffraction plane shifts opposite to the (001) layered basal diffraction plane, but changed an order of magnitude smaller than the (001) plane, by only 0.22° 2θ. During the charging process the (020) shifted to higher 2θ angle (23.16° 2θ) and smaller d-spacing (1.850 Å), due to the increased oxidation state of V and shorter V-O bond distance. Upon discharging the (020) peak shifts to lower 2θ (22.94° 2θ) and larger d-spacing (1.867 Å) as the V is reduced and the V-O bond distance increases.

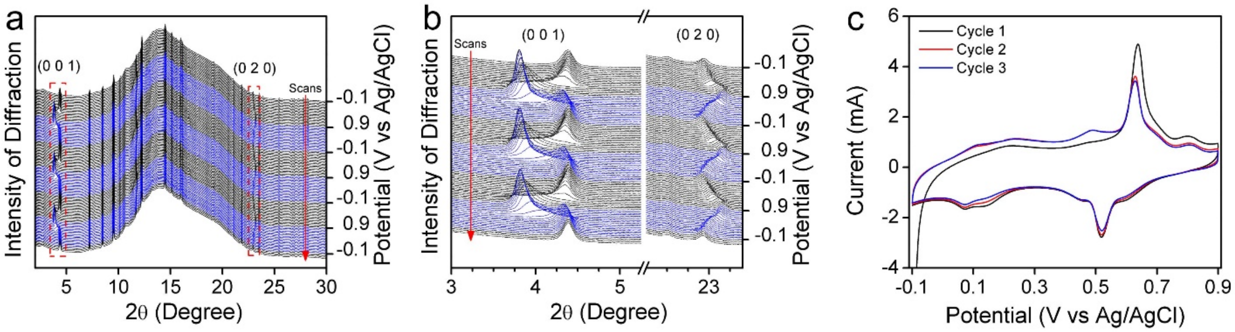

Supplementary Figure S18. *In situ* XRD of ordered KVO. (**a**) Full spectra of *in situ* XRD showing the large background from the aqueous 3 M KCl electrolyte. (**b**) close up of the selected regions showing the evolution of the (001) and (020) peaks with potential through three cycles. (**c**) Three cycle CV measurement at 1 mV/s in a 3 M KCl electrolyte conducted while collecting the *in situ* XRD spectra.

Supplementary Tables

Supplementary Table S1. X-ray/Neutron PDF results of the disordered KVO nanosheets from **Figure 1d&e.**

Cell Symmetry: Monoclinic C 2/m

a	b	c	α	β	γ
11.748	3.629	11.371	90	105.7	90

Atom	Label	X	Y	Z	Occ	U11	U22	U33	U12	U13	U23
**K**	K1	0.1540	0	0.4854	0.22	0.006	0.006	0.006	0	0	0
**V**	V1	0.9021	0	0.8730	0.74	0.009	0.005	0.065	0	0.014	0
**V**	V2	0.1884	0	0.8599	1	0.011	0.005	0.015	0	0.007	0
**O**	O1	0.8673	0.5	0.8923	0.72	0.049	0.013	0.009	0	0.021	0
**O**	O2	0.7345	0	0.9095	0.80	0.021	0.012	0.041	0	0.009	0
**O**	O3	0.0676	0	0.9133	1	0.031	0.018	0.024	0	0.012	0
**O**	O4	0.8616	0	0.7068	1	0.024	0.041	0.034	0	0.025	0
**O**	O5	0.1418	0	0.7160	0.87	0.026	0.048	0.010	0	0.000	0
**O**	Ow1	0.4276	0	0.5532	0.41	0.020	0.020	0.020	0	0.009	0
**O**	Ow2	0.3208	0	0.4899	0.41	0.020	0.020	0.020	0	0.010	0

Supplementary Table S2. Neutron PDF of the partially hydrated (not soaked in DI H_2O for two weeks) disordered KVO nanosheets results from **Figure 3b.**

Cell Symmetry: Monoclinic C 2/m

a	b	c	α	β	γ
11.990	3.579	11.498	90	105.8	90

Atom	Label	X	Y	Z	Occ	U11	U22	U33	U12	U13	U23
**K**	K1	0.2199	0	0.5379	0.22	0.009	0.009	0.009	0	0	0
**V**	V1	0.8911	0	0.9113	0.74	0.010	0.010	0.010	0	0	0
**V**	V2	0.2093	0	0.9016	1.00	0.005	0.005	0.005	0	0	0
**O**	O1	0.8793	0.5	0.8777	0.66	0.016	0.044	0.070	0	0.004	0
**O**	O2	0.7283	0	0.9063	0.76	0.028	0.041	0.021	0	0.005	0
**O**	O3	0.0506	0	0.9044	0.93	0.020	0.058	0.026	0	0.017	0
**O**	O4	0.8382	0	0.7035	1.00	0.088	0.046	0.013	0	0.023	0
**O**	O5	0.1554	0	0.7257	0.80	0.017	0.137	0.016	0	-0.003	0
**O**	Ow1	0.4213	0	0.5682	0.23	0.022	0.022	0.022	0	0	0
**O**	Ow2	0.3146	0	0.4918	0.23	0.022	0.022	0.022	0	0	0

Supplementary Notes

Calculation of capacitance in half-cell.
The CV measurements were conducted in 1M KCl electrolyte at scan rates from 5 to 200 mV s^{-1} within a potential range from – 0.1 V to 0.9 V (vs. Ag/AgCl). The mass-specific capacitance (C_{MS}) from CV measurement in half-cell was calculated from:

$$C_{MS} = \frac{i}{(dV/dt)*m} = \int_{t_0}^{t_F} \frac{i}{\Delta V*m} dt \qquad \text{Supplementary Eq. (1)}$$

Where i (A) is the measured current at certain time of t (s), m (g) is the mass of active material loaded on working electrode, ΔV (V) is potential window, t_0 (s) and t_F (s) are respective times at the initial potential and the final potential.

Electrochemical full cell calculations.
The cell capacitance C_{cell} (F), cell mass-specific capacitance C_{MS} (F g^{-1}), electrode mass-specific capacitance $C_{MS(electrode)}$ (F g^{-1}), discharge cell capacity by mass $C_{discharge}$ (mAh g^{-1}), discharge electrode capacity by mass $C_{discharge(electrode)}$ (mAh g^{-1}), specific energy E_{MS} (Wh kg^{-1}), specific power P_{MS} (W kg^{-1}) and coulombic efficiency (η) and energy efficiency (γ) were calculated by following equations:

Cell capacitance: $C_{cell} = \frac{it}{U}$ Supplementary Eq. (2)

Cell mass-specific capacitance: $C_{MS} = \frac{it}{U*M} = \frac{it}{U*2m}$ Supplementary Eq. (3)

Electrode mass-specific capacitance: $C_{MS(electrode)} = 4C_{MS} = \frac{2it}{U*M/2} = \frac{2it}{U*m}$
Supplementary Eq. (4)

Discharge cell capacity by mass: $C_{discharge} = \frac{it}{3.6*M} = \frac{it}{3.6*2m}$ Supplementary Eq. (5)

Discharge electrode capacity by mass:

$C_{discharge(electrode)} = 4C_{discharge} = \frac{2it}{3.6*M/2} = \frac{2it}{3.6*m}$ Supplementary Eq. (6)

Coloumbic efficiency: $\eta = \frac{Q_{discharge}}{Q_{charge}} = \frac{it_{discharge}}{it_{charge}} = \frac{t_{discharge}}{t_{charge}}$ Supplementary Eq. (7)

Where i (A) is the applied constant current, t (s) is discharge time of the cell device, U (V) is potential window, M (g) is the total mass of active materials on both electrodes and m (g) is the mass of active materials on one electrode.
